# The InBIO Barcoding Initiative Database: DNA barcodes of Portuguese Diptera 02 - Limoniidae, Pediciidae and Tipulidae

**DOI:** 10.3897/BDJ.9.e69841

**Published:** 2021-09-24

**Authors:** Sónia Ferreira, Pjotr Oosterbroek, Jaroslav Starý, Pedro Sousa, Vanessa A. Mata, Luis P da Silva, Joana Paupério, Pedro Beja

**Affiliations:** 1 CIBIO-InBIO, Centro de Investigação em Biodiversidade e Recursos Genéticos, Universidade do Porto, Vila do Conde, Portugal CIBIO-InBIO, Centro de Investigação em Biodiversidade e Recursos Genéticos, Universidade do Porto Vila do Conde Portugal; 2 Naturalis Biodiversity Center, Leiden, Netherlands Naturalis Biodiversity Center Leiden Netherlands; 3 Olomouc-Nedvězí & Silesian Museum, Nádražní okruh 31, CZ-746 01 Opava, Olomouc, Czech Republic Olomouc-Nedvězí & Silesian Museum, Nádražní okruh 31, CZ-746 01 Opava Olomouc Czech Republic; 4 CIBIO-InBIO, Centro de Investigação em Biodiversidade e Recursos Genéticos, Instituto Superior de Agronomia, Universidade de Lisboa, Tapada da Ajuda, 1349-017, Vila do Conde, Portugal CIBIO-InBIO, Centro de Investigação em Biodiversidade e Recursos Genéticos, Instituto Superior de Agronomia, Universidade de Lisboa, Tapada da Ajuda, 1349-017 Vila do Conde Portugal

**Keywords:** Diptera, Limoniidae, Pediciidae, Tipulidae, occurrence records, Portugal, DNA barcode, COI

## Abstract

**Background:**

The InBIO Barcoding Initiative (IBI) Diptera 02 dataset contains records of 412 crane fly specimens belonging to the Diptera families: Limoniidae, Pediciidae and Tipulidae. This dataset is the second release by IBI on Diptera and it greatly increases the knowledge on the DNA barcodes and distribution of crane flies from Portugal. All specimens were collected in Portugal, including six specimens from the Azores and Madeira archipelagos. Sampling took place from 2003 to 2019. Specimens have been morphologically identified to species level by taxonomists and belong to 83 species in total. The species, represented in this dataset, correspond to about 55% of all the crane fly species known from Portugal and 22% of crane fly species known from the Iberian Peninsula. All DNA extractions and most specimens are deposited in the IBI collection at CIBIO, Research Center in Biodiversity and Genetic Resources.

**New information:**

Fifty-three species were new additions to the Barcode of Life Data System (BOLD), with another 18 species' barcodes added from under-represented species in BOLD. Furthermore, the submitted sequences were found to cluster in 88 BINs, 54 of which were new to BOLD. All specimens have their DNA barcodes publicly accessible through BOLD online database and its collection data can be accessed through the Global Biodiversity Information Facility (GBIF). One species, *Gonomyiatenella* (Limoniidae), is recorded for the first time from Portugal, raising the number of crane flies recorded in the country to 145 species.

## Introduction

Portugal is part of the Mediterranean hotspot of biodiversity, yet Portuguese biodiversity remains poorly studied and genetic data are scarcer still. To tackle this problem, the Research Network in Biodiversity and Evolutionary Biology (InBIO) created the InBIO Barcoding Initiative (IBI), making use of in-house High Throughput Sequencing knowledge to construct a reference collection of morphologically identified Portuguese specimens and corresponding DNA barcodes (Ferreira et al. 2018). Invertebrates, especially insects, were given priority in the IBI due to their share of overall biodiversity and importance in ecosystems functioning (e.g. Weisser and Siemann 2004, Losey and Vaughan 2006, Mata et al. 2016, da Silva et al. 2019) and due to the lack of available DNA barcodes in public databases representative of Portuguese Invertebrates (e.g. Corley and Ferreira 2017, Corley et al. 2017, Ferreira et al. 2019, Weigand et al. 2019).

DNA barcoding is a molecular biology method for species identification that relies on the comparison of a short mitochondrial DNA sequence of interest to a library of sequences with known species identity (Hebert et al. 2003). The construction of comprehensive reference libraries is therefore essential and these require the morphological identification of vouchers by expert taxonomists (Kress et al. 2015, Ferreira et al. 2018). DNA barcoding has expanded beyond single organism and species identification, to broader metabarcoding studies (Porter and Hajibabaei 2020). DNA barcodes are now a ubiquitous tool in ecological and biological conservation studies, as well as, for example, in forensic applications (Pečnikar and Buzan 2013, Kress et al. 2015, DeSalle and Goldstein 2019).

The order Diptera is one of the most diverse, widespread and common of the holometabolic insects, having more than 158,000 described species (Pape et al. 2009, Evenhuis and Pape 2021). Within Diptera, the crane flies (Tipuloidea) are further classified into four families, Cylindrotomidae, Limoniidae, Pediciidae and Tipulidae (Starý 1992; but see Petersen et al. 2010 and Starý 2021) and are one of the most diverse groups, with over 15,630 recognised species (Oosterbroek 2021). Adult Tipuloidea can superficially resemble mosquitoes, with their slender bodies and long antennae, wings, legs and abdomen, but can be identified by the presence of two complete anal veins in the wings, a V-shaped transverse suture on the mesothorax and the absence of ocelli (de Jong et al. 2007). Larvae of Tipuloidea are mainly identified by the presence of a hemicephalous, retractible head capsule ocelli (de Jong et al. 2007). Larvae of most species are found in aquatic habitats, from fast-flowing streams to brackish water or in semi-aquatic habitats, such as organic sludge along the edge of water bodies or saturated mosses and hepatics. Those that are terrestrial are mostly still found in humid habitats, like leaf-litter (Pritchard 1983, de Jong et al. 2007), although a few also live in dry soils. Contrary to immature forms, all adult Tipuloidea, mostly short-lived after emergence, are terrestrial (Pritchard 1983, de Jong et al. 2007), while in the larvae stage, most species feed on algae or decaying plant material and associated microflora and some groups also feed on mosses and hepatics, though several Limnophilinae and Pediciinae larvae are predatory (Pritchard 1983, de Jong et al. 2007). Most species do not feed after reaching adulthood, although adults generally drink water to offset body evaporation (Pritchard 1983, de Jong et al. 2007). A few species are known to be important crop pests, as their larvae, when in large numbers, can damage crops by feeding on their roots or seedlings (Alford 2012, Alford 2014, Blackshaw and Coll 1999, de Jong et al. 2007). Furthermore, species of Tipuloidea play important ecological roles in several ecosystems being well known components of bird and bat diets (e.g. Alford 2012, Alford 2014, Buchanan et al. 2006, Krüger et al. 2013, Rhymer et al. 2012, Vaughan 1997, Wilson et al. 1999).

In Portugal, the knowledge on Tipuloidea is still very incomplete. Of the four families that compose the Tipuloidea, only the Cylindrotomidae has not so far been recorded for the country, although it is known from Spain (Oosterbroek et al. 2020, Oosterbroek 2021). Recently, 33 new species were added to the Portuguese species list, raising its total to 149 (Eiroa and Báez 2002a, Eiroa and Báez 2002b, Oosterbroek et al. 2020, Oosterbroek 2021, Kolcsár et al. 2021). This is certainly an underestimate as 376 species are already known from the Iberian Peninsula (Oosterbroek et al. 2020). Furthermore, the distribution and ecology of the Portuguese crane flies are also poorly known.

The IBI Diptera 02 dataset contains records of 412 specimens of crane flies collected in Portugal, all morphologically identified to species level, for a total of 83 species, two of which were further identified to subspecies level. This dataset is part of the ongoing IBI database public releases in both the Global Biodiversity Information Facility (GBIF) and the Barcode of Life Data System (BOLD) (e.g. Ferreira et al. 2020a, Ferreira et al. 2020b).

## General description

### Purpose

This dataset aims to provide the second contribution to a DNA barcode sequences library for Portuguese Diptera. It covers the three families of Tipuloidea known from Portugal. This library aims to advance DNA-based species identification from regular molecular studies and new DNA-metabarcoding studies. It presents also an important resource for taxonomic research on Portuguese crane flies and its distribution.

### Additional information

A total of 412 specimens of crane flies were collected and DNA barcoded (Suppl. materials 1, 2). Fig. 1 illustrates examples of the diversity of species that are part of the dataset of distribution data and DNA barcodes of Portuguese Diptera 02. All sequences of cytochrome c oxidase I (COI) DNA barcodes are 658 bp long. This dataset contributes significantly to the representation of both species and genetic diversity of Tipuloidea in public libraries. Of the 83 taxa barcoded, 53 (64%) are new to the DNA barcode database BOLD at the moment of the release (marked with * in Taxa field of Table 1). Moreover, the species *Gonomyiatenella* (Meigen, 1818) is recorded for the first time for Portugal. Eighteen species in the dataset (22%) were previously represented in BOLD with less than 10 publicly available DNA barcode sequences at the moment of the release (marked with '' in Species field of Table 1). The submitted sequences were found to cluster in 88 BINs in BOLD (Barcode Index Number, Ratnasingham and Hebert 2013), 54 of which were new to BOLD. Of the submitted sequences, *Tipulaintermedia* Eiroa, 1990 and *Tipulalateralis* Meigen, 1804 share the same BIN in BOLD. Moreover, the generated sequence of *Nephrotomasuturaliswulpiana* (Bergroth, 1888) shared BIN with sequences of *Nephrotomaferruginea* (Fabricius, 1805) from the United States of America, where both species are native. These two species are very closely related and are frequenly misidentified. Differences between the two species have been outlined in the revision of the Nearctic non-dorsalis species (Oosterbroek 1984). The two former records from Portugal are discussed in detail and figured in Hancock et al. 2016. The generated sequence of *Molophilustestaceus* Lackschewitz, 1940 shares the BIN with *Molophilusgriseus* (Meigen, 1804) from Finland and with a sequence of a specimen identified as Molophiluscf.ochraceus (Meigen, 1818) from Norway. The generated sequences of *Pseudolimnophilaebullata* Stary, 1982 share the BIN with a sequence of *Pseudolimnophilasepium* (Verrall, 1886) from Estonia. In addition, the generated sequences of *Nephrotomasubmaculosa* Edwards, 1928 clustered in two BINs, one of which also harbours sequences of *Nephrotomaflavescens* (Linnaeus, 1758) from Germany and Finland. The identifications of *Nephrotomasuturaliswulpiana* and the *Nephrotomasubmaculosa* specimens clustering with *Nephrotomaflavescens* were double-checked by Herman de Jong (Naturalis Biodiversity Center, Leiden, The Netherlands).

## Project description

### Title

The InBIO Barcoding Initiative Database: DNA barcodes of Portuguese Diptera 02 - Limoniidae, Pediciidae and Tipulidae

### Personnel

Pedro Beja (project coordinator), Sónia Ferreira (taxonomist and IBI manager), Joana Paupério (IBI manager), Pedro Sousa (project technician), Vanessa Mata (contributor) and Luis P da Silva (contributor), all affiliated to CIBIO-InBIO; Pjotr Oosterbroek (taxonomist), affiliated to Naturalis and Jaroslav Starý (taxonomist), affiliated to Olomouc-Nedvězí & Silesian Museum.

### Study area description

**Figure 2.**
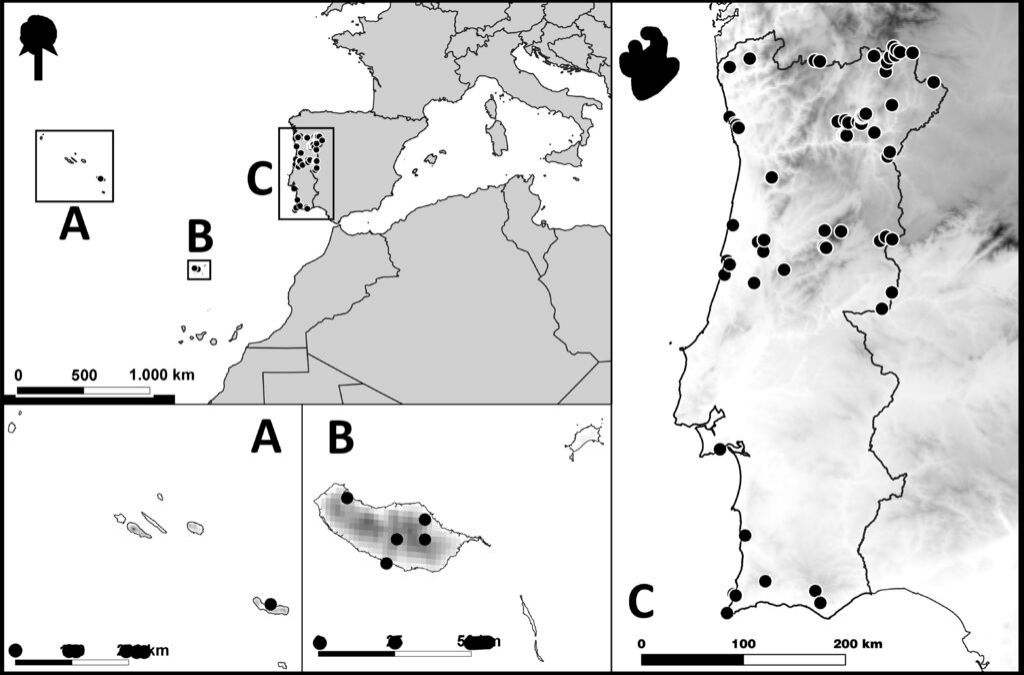
Map of the localities where crane fly samples were collected in Portugal. **A.** Azores archipelago; **B.** Madeira archipelago (part); **C.** Continental Portugal.

Portugal, including the Autonomous Regions of the Azores and of Madeira (Fig. 2).

### Design description

Tipuloidea specimens were collected in the field, morphologically identified and DNA barcoded.

### Funding

This project was funded by European Union’s Horizon 2020 Research and Innovation Programme under grant agreement No 668981 and by project PORBIOTA - Portuguese E-Infrastructure for Information and Research on Biodiversity (POCI-01-0145-FEDER-022127), supported by Operational Thematic Program for Competitiveness and Internationalization (POCI), under the PORTUGAL 2020 Partnership Agreement, through the European Regional Development Fund (FEDER). The fieldwork benefited from EDP Biodiversity Chair, the project “Promoção dos serviços de ecossistemas no Parque Natural Regional do Vale do Tua: Controlo de Pragas Agrícolas e Florestais por Morcegos”, funded by the Agência de Desenvolvimento Regional do Vale do Tua and includes research conducted at the Long Term Research Site of Baixo Sabor (LTER_EU_PT_002). LPdS and SF were supported by individual research contracts (CEECIND/02064/2017, 2020.03526.CEECIND), funded by FCT. The work of JS was funded by the Ministry of Culture of the Czech Republic through institutional financing of long-term conceptual development of the Silesian Museum Research Institution (MK000100595).

## Sampling methods

### Study extent

Portugal, including the Autonomous Regions of the Azores and of Madeira.

### Sampling description

The studied material was collected in 83 different localities from Portugal, 77 from continental Portugal and six from the Autonomous Regions of the Azores and of Madeira. The Bragança District was the most heavily sampled (21% of total specimens) and where most species were recorded, with almost half of the species (41%) in the dataset found there (Fig. 2, Table 2). Sampling was conducted between 2003 and 2019, although the vast majority of specimens were collected in 2018 (32%) and 2019 (50%). Specimens were collected by direct search and individual netting of specimens, by sweeping the vegetation or were directly collected at light traps (using both UV and mercury vapour lights) and stored in 96% ethanol for downstream molecular analysis, unless stated otherwise.

DNA extraction and sequencing followed the general pipeline in use by the IBI. Genomic DNA was extracted from leg tissue using the EasySpin Genomic DNA Tissue Kit (Citomed) according to the manufacturer’s protocol. The cytochrome c oxidase I (COI) barcoding fragment was then amplified as two overlapping fragments (LC and BH), using two sets of primers: LCO1490 (Folmer et al. 1994) + Ill_C_R (Shokralla et al. 2015) and Ill_B_F (Shokralla et al. 2015) + HCO2198 (Folmer et al. 1994), respectively. The COI barcode (Folmer region) was then sequenced in a MiSeq benchtop system. OBITools (https://git.metabarcoding.org/obitools/obitools) was used to process the initial sequences which were then assembled into a single 658 bp fragment using Geneious 9.1.8. (https://www.geneious.com).

### Quality control

All DNA barcodes sequences were compared against the BOLD database and the top 99 hits were inspected to detect possible problems arising from contaminations or misidentifications. The data were checked for errors and inconsistencies with OpenRefine 3.4 (http://openrefine.org) before submission to GBIF.

### Step description

1. Specimens were collected in 83 different Portuguese localities. Fieldwork was carried out between 2003 and 2019, with 82% of the records made in the years 2018 and 2019.
2. Specimens were collected during fieldwork by direct search and individual netting of specimens, by sweeping the vegetation or were directly collected at light traps (using both UV and mercury vapour lights) and preserved in 96% alcohol. The majority of captured specimens were deposited in the IBI reference collection at CIBIO (Research Center in Biodiversity and Genetic Resources).
3. All specimens were morphologically identified using the available literature, except seven that were identified using the BOLD Identification Engine. For some specimens, it was necessary to prepare and then exam their terminalia.
4. All specimens were DNA barcoded. To sequence the 658 bp COI DNA barcode fragment, one leg was removed from each individual, DNA was extracted and then amplified. All DNA extracts were deposited in the IBI collection.
5. All sequences in the dataset were submitted to BOLD and GenBank databases and, to each sequenced specimen, the morphological identification was contrasted with the results of the BLAST of the newly-generated DNA barcodes in the BOLD Identification Engine.
6. Prior submission to GBIF, data were checked for errors and inconsistencies with OpenRefine 3.4 (http://openrefine.org/).

## Geographic coverage

### Description

Continental Portugal, Autonomous Regions of the Azores and of Madeira.

### Coordinates

32.65 and 41.97 Latitude; -25.51 and -6.34 Longitude.

## Taxonomic coverage

### Description

**Figure 3.**
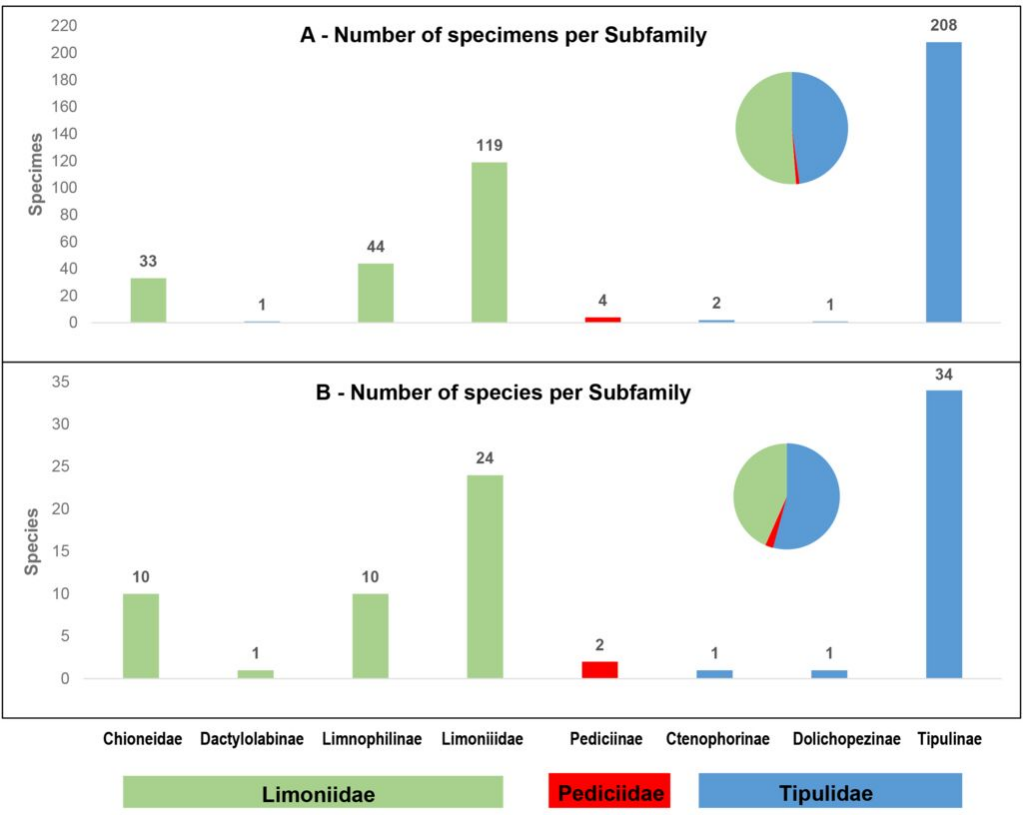
Number of specimens (**A**) and species (**B**), per Tipuloidea (Diptera) family and subfamily present in the dataset. Pie chart depicts family only data.

The dataset is composed of data relating to 412 specimens of Diptera, all from the Tipuloidea superfamily. All specimens were morphologically identified to species or subspecies level by Pjotr Oosterbroek and/or Jaroslav Starý, except for seven specimens identified using the BOLD Identification Engine. In total, 83 species and two subspecies are represented in the dataset. These species belong to three families, Limoniidae, Pediciidae and Tipulidae. Limoniidae and Tipulidae account for similar numbers of collected specimens, 197 (48%) and 211 (51%) (Fig. 3A), respectively, although Limoniidae have a higher proportion of recorded species in the dataset (45 species - 54% of the total) when compared with Tipulidae (36 species - 43%) (Fig. 3B). At the subfamily level, Tipulinae and Limoniinae represented the most collected specimens (50% and 29%, Fig. 3A) and also the highest number of recorded species in the dataset (41% and 29%, Fig. 3B). The species, represented in this dataset, correspond to about 55% of all the crane fly species known from Portugal (52% Limoniidae, 66% Pediciidae and 57% Tipulidae) and 22% of crane fly species known from the Iberian Peninsula.

### Taxa included

**Table:** 

Rank	Scientific Name	
kingdom	Animalia	
phylum	Arthropoda	
class	Insecta	
order	Diptera	
superfamily	Tipuloidea	
family	Limoniidae	
family	Pediciidae	
family	Tipulidae	
subfamily	Chioneinae	
subfamily	Ctenophorinae	
subfamily	Dactylolabinae	
subfamily	Dolichopezinae	
subfamily	Limnophilinae	
subfamily	Limoniinae	
subfamily	Pediciinae	
subfamily	Tipulinae	

## Temporal coverage

**Data range:** 2003-9-08 – 2019-10-04.

### Notes

The sampled material was collected in the period from 8 September 2003 to 14 October 2019.

## Usage licence

### Usage licence

Other

### IP rights notes

Creative Commons Attribution 4.0 International (CC BY 4.0)

## Data resources

### Data package title

The InBIO Barcoding Initiative Database: DNA barcodes of Portuguese Diptera 02 - Limoniidae, Pediciidae and Tipulidae

### Resource link

dx.doi.org/10.5883/DS-IBIDP02

### Number of data sets

1

### Data set 1.

#### Data set name

DS-IBIDP02 IBI Diptera 02

#### Data format

dwc, xml, tsv, fasta

#### Number of columns

37

#### Download URL

http://www.boldsystems.org/index.php/Public_SearchTerms?query=DS-IBIDP02

#### Description

The InBIO Barcoding Initiative Database: DNA barcodes of Portuguese Diptera 02 - Limoniidae, Pediciidae and Tipulidae dataset can be downloaded from the Public Data Portal of BOLD (dx.doi.org/10.5883/DS-IBIDP02) in different formats (data as dwc, xml or tsv and sequences as fasta files). BOLD users can also log-in and access the dataset through the Workbench platform of BOLD. All records are also discoverable within BOLD, using the platform search function.

The InBIO Barcoding Initiative will continue to sequence crane flies and other Diptera for the BOLD database, with the ultimate objective of achieving a comprehensive coverage of the Portuguese fauna. The version of the dataset, at the time of writing the manuscript, is included as Suppl. materials 1, 2, 3 in the form of two text files with specimen data, as downloaded from BOLD and from GBIF (the latter in Darwin Core Standard format) and one fasta file containing all sequences as downloaded from BOLD.

The BOLD database is not completely compliant with the Darwin Core Standard (DCS) format and, as such, the Darwin Core formatted file (dwc) downloaded from the BOLD platform is not strictly DCS formatted. For a correctly DCS formatted file, see http://ipt.gbif.pt/ipt/resource?r=ibi_diptera_02&amp;v=1.6 (Suppl. material 2).

Column labels below follow the labels downloaded in the tsv file downloaded from BOLD. Columns with no content in our dataset are left out in the list below.

**Data set 1.** 

Column label	Column description
processid	Unique identifier for the sample
sampleid	Identifier for the sample being sequenced, i.e. IBI catalogue number at Cibio-InBIO, Porto University. Often identical to the "Field ID" or "Museum ID"
recordID	Identifier for specimen assigned in the field
catalognum	Catalogue number
fieldnum	Field number
institution_storing	The full name of the institution that has physical possession of the voucher specimen
bin_uri	Barcode Index Number system identifier
phylum_taxID	Phylum taxonomic numeric code
phylum_name	Phylum name
class_taxID	Class taxonomic numeric code
class_name	Class name
order_taxID	Order taxonomic numeric code
order_name	Order name
family_taxID	Family taxonomic numeric code
family_name	Family name
subfamily_taxID	Subfamily taxonomic numeric code
subfamily_name	Subfamily name
genus_taxID	Genus taxonomic numeric code
genus_name	Genus name
species_taxID	Species taxonomic numeric code
species_name	Species name
subspecies_taxID	Subspecies taxonomic numeric code
subspecies_name	Subspecies name
identification_provided_by	Full name of primary individual who assigned the specimen to a taxonomic group
identification_method	The method used to identify the specimen
voucher_status	Status of the specimen in an accessioning process (BOLD controlled vocabulary)
tissue_type	A brief description of the type of tissue or material analysed
collectors	The full or abbreviated names of the individuals or team responsible for collecting the sample in the field
lifestage	The age class or life stage of the specimen at the time of sampling
sex	The sex of the specimen
lat	The geographical latitude (in decimal degrees) of the geographic centre of a location
lon	The geographical longitude (in decimal degrees) of the geographic centre of a location
elev	Elevation of sampling site (in metres above sea level)
country	The full, unabbreviated name of the country where the organism was collected
province_state	The full, unabbreviated name of the Province ("Distrito" in Portugal) where the organism was collected
region	The full, unabbreviated name of the Municipality ("Concelho" in Portugal) where the organism was collected
exactsite	Additional name/text description regarding the exact location of the collection site relative to a geographic relevant landmark

## Supplementary Material

A802F048-BEAF-5AD2-8E90-7C6EFAFA1311 10.3897/BDJ.9.e69841.suppl1 Supplementary material 1 IBI - Diptera 02 library - Specimen details Data type Specimen data records Brief description The file includes information about all records in BOLD for the IBI - Diptera 02 library. It contains collecting and identification data. The data are as downloaded from BOLD in the tsv format, without further processing. File: oo_570642.txt https://binary.pensoft.net/file/570642 Pedro Sousa, Pjotr Oosterbroek, Jaroslav Stary, Vanessa A Mata, Luis P. da Silva, Pedro Beja, Sónia Ferreira

E9FB060C-59A2-54F9-AFC9-FE49E7E7F1E9 10.3897/BDJ.9.e69841.suppl2 Supplementary material 2 IBI - Diptera 02 library - Specimen details - Darwin Core Standard Data type Specimen data records in the Darwin Core Standard format Brief description The file includes information about all records in GBIF for the IBI - Diptera 02 library. It contains collecting and identification data. The data are as downloaded from GBIF, without further processing. File: oo_570646.txt https://binary.pensoft.net/file/570646 Pedro Sousa, Pjotr Oosterbroek, Jaroslav Stary, Vanessa A Mata, Luis P. da Silva, Pedro Beja, Sónia Ferreira

8A163908-CBCB-56C6-AC95-022D6A10EE03 10.3897/BDJ.9.e69841.suppl3 Supplementary material 3 IBI- Diptera 02 library - DNA sequences Data type Specimen genomic data, DNA sequences Brief description COI sequences in fasta format. Each sequence is identified by the BOLD ProcessID, species name, genetic marker name and GenBank accession number, all separated by a vertical bar. The data are as downloaded from BOLD. File: oo_546735.fas https://binary.pensoft.net/file/546735 Pedro Sousa, Joana Paupério, Pedro Beja, Sónia Ferreira

## Figures and Tables

**Figure 1a.**
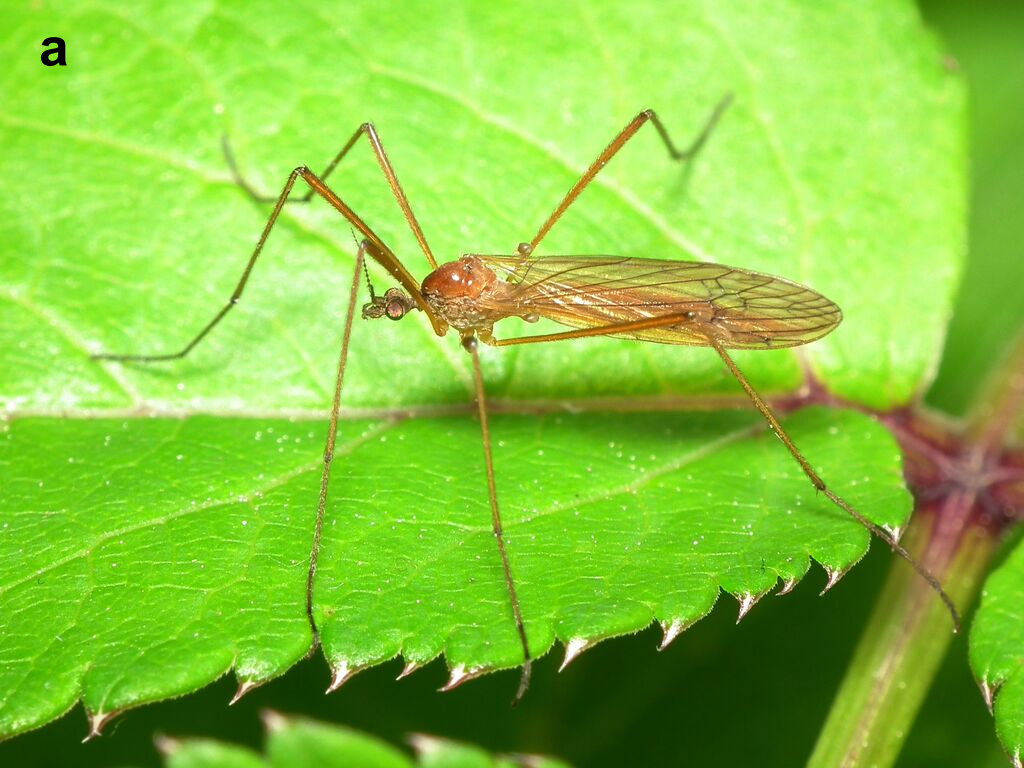
*Phylidoreaferruginea* (Meigen, 1818) - BIN URI BOLD:ABW4832

**Figure 1b.**
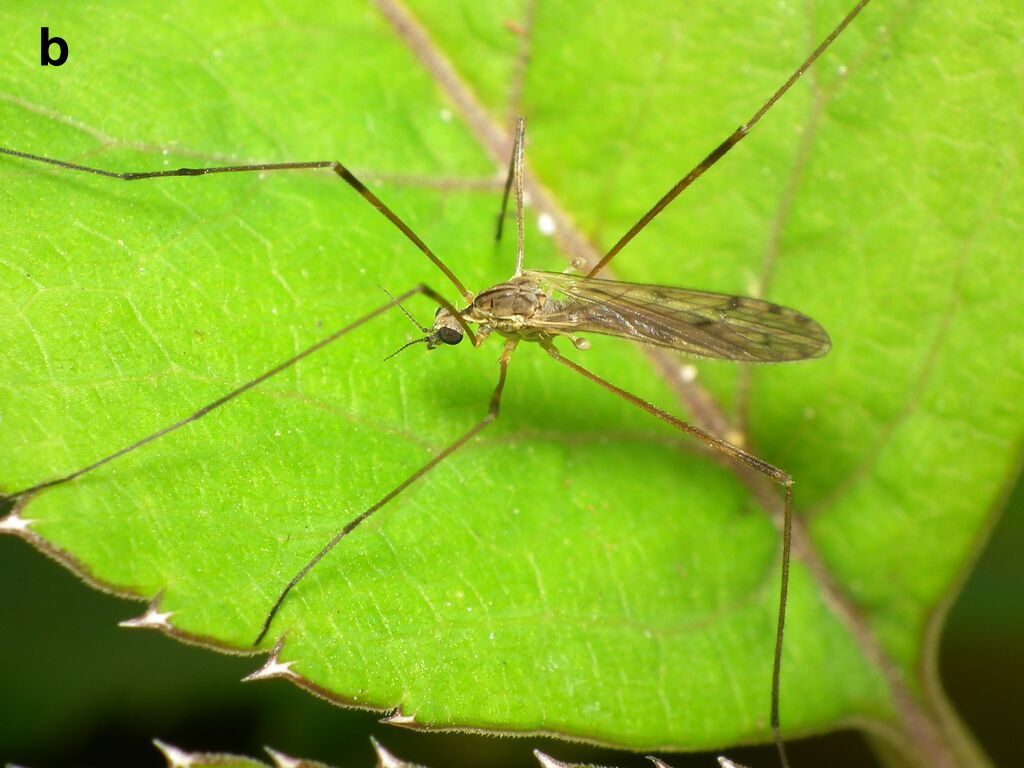
*Symplectastictica* (Meigen, 1818) - BIN URI BOLD:ADN4631

**Figure 1c.**
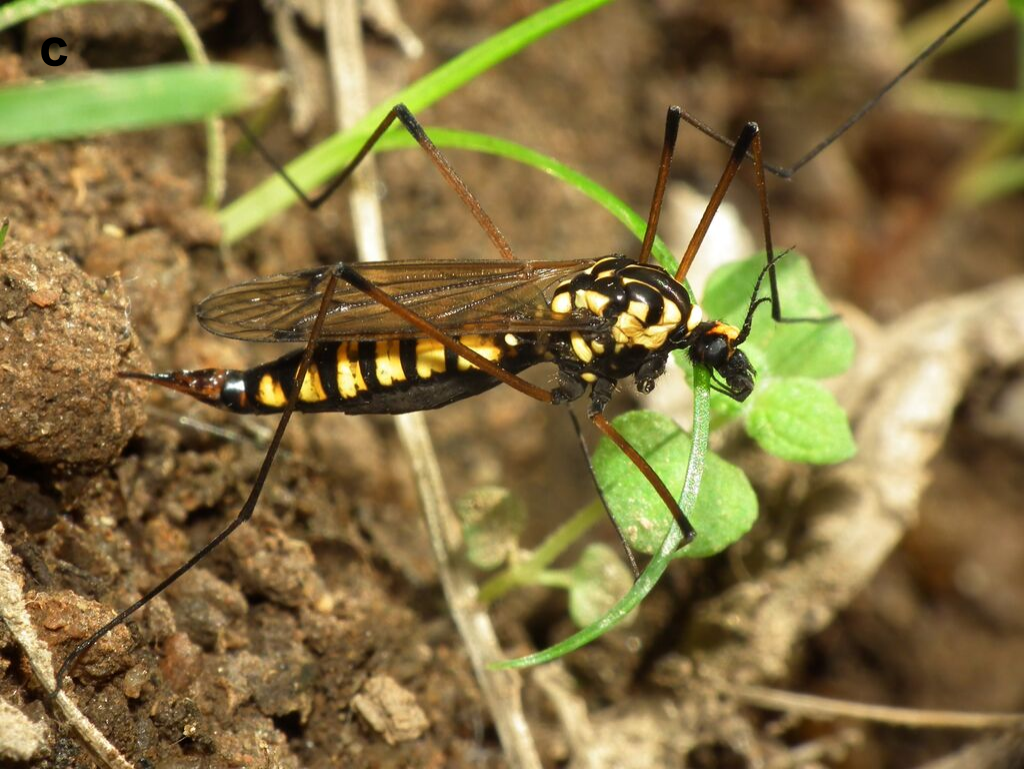
*Nephrotomaluteata* (Meigen, 1818) - BIN URI: BOLD:ADW1410

**Figure 1d.**
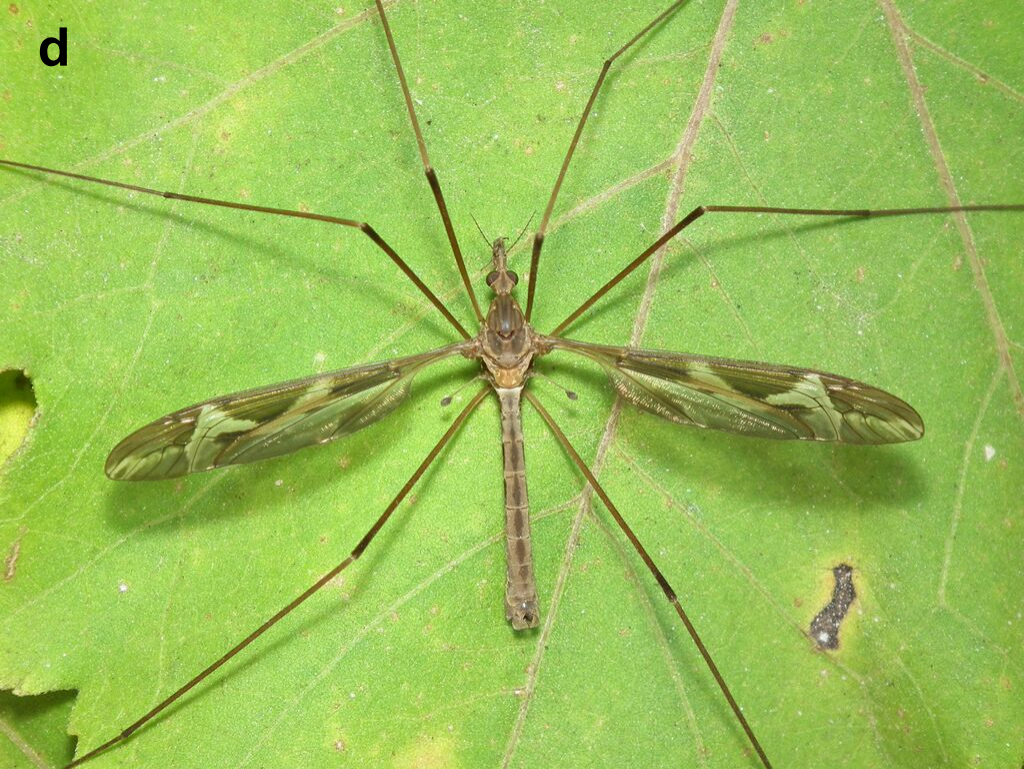
*Tipularepanda* Loew, 1864 - BIN URI: BOLD:AEC7761

**Table 1.** List of taxa that were collected and DNA barcoded within this project. In column Taxa: * - Indicates taxa without a DNA barcode prior to this study; '' - indicates taxa with less than 10 sequences available prior to this study.

**Family (Subfamily)**	**Taxa**	**IBI code A**	**BOLD code**	**BOLD BIN**	**GenBank**
Limoniidae (Chioneinae)	*Erioconopadiuturna* (Walker, 1848) "	INV06209, INV08150	IBIDP258-19, IBIDP446-19	BOLD:ADZ3321	MZ196613, MZ196520
Limoniidae (Chioneinae)	*Eriopterafuscipennis* Meigen, 1818 *	INV08142	IBIDP438-19	BOLD:AEC7071	MZ196632
Limoniidae (Chioneinae)	*Gonomyiatenella* (Meigen, 1818) "	INV08655	IBIDP644-20	BOLD:ABV4194	MZ196605
Limoniidae (Chioneinae)	*Idiocerasziladyi* (Lackschewitz, 1940) *	INV07222, INV07671, INV07679, INV08144, INV08393, INV08394, INV08395, INV08396	IBIDP331-19, IBIDP338-19, IBIDP339-19, IBIDP440-19, IBIDP474-19, IBIDP475-19, IBIDP476-19, IBIDP477-19	BOLD:AEC9216	MZ196587, MZ196919, MZ196788, MZ196902, MZ196747, MZ196607, MZ196872, MZ196585
Limoniidae (Chioneinae)	*Molophilusbaezi* Theowald, 1981 *	INV08355	IBIDP450-19	BOLD:AEC9561	MZ196837
Limoniidae (Chioneinae)	*Molophilusflavus* Goetghebuer, 1920 "	INV06375	IBIDP283-19	BOLD:AEC9823	MZ196640
Limoniidae (Chioneinae)	*Molophilustestaceus* Lackschewitz, 1940 *	INV10269	IBIDP810-20	BOLD:ABV9683	MZ196639
Limoniidae (Chioneinae)	*Symplectahybrida* (Meigen, 1804)	INV07037, INV08356	IBIDP323-19, IBIDP451-19	BOLD:AAZ4292	MZ196555, MZ196715
Limoniidae (Chioneinae)	*Symplectapilipes* (Fabricius, 1787)	INV06218, INV06499, INV06992, INV08153, INV08592, INV08597, INV08599, INV09687, INV09693, INV09695, INV09709, INV10185, INV10192, INV10310	IBIDP260-19, IBIDP294-19, IBIDP321-19, IBIDP449-19, IBIDP636-20, IBIDP637-20, IBIDP639-20, IBIDP766-20, IBIDP767-20, IBIDP768-20, IBIDP769-20, IBIDP783-20, IBIDP785-20, IBIDP830-20	BOLD:ACO3207	MZ196843, MZ196778, MZ196859, MZ196685, MZ196601, MZ196804, MZ196593, MZ196776, MZ196833, MZ196539, MZ196525, MZ196787, MZ196858, MZ196908
Limoniidae (Chioneinae)	*Symplectastictica* (Meigen, 1818)	INV06513, INV08125	IBIDP297-19, IBIDP421-19	BOLD:ADN4631	MZ196807, MZ196840
Limoniidae (Dactylolabinae)	*Dactylolabissexmaculata* (Macquart, 1826) *	INV04587	IBIDP193-19	BOLD:ADW0650	MZ196744
Limoniidae (Limnophilinae)	*Austrolimnophilaanalis* (Santos Abreu, 1923) *	INV08357	IBIDP452-19	BOLD:AEC6671	MZ196681
Limoniidae (Limnophilinae)	*Austrolimnophilalatistyla* Starý, 1977 *	INV07797, INV08145, INV08440, INV08507, INV08531, INV08535, INV08538, INV08542, INV08549, INV08551, INV08554, INV08556, INV08557, INV08608, INV08661, INV08775, INV08817, INV09017, INV10347	IBIDP415-19, IBIDP441-19, IBIDP594-20, IBIDP619-20, IBIDP622-20, IBIDP624-20, IBIDP625-20, IBIDP626-20, IBIDP628-20, IBIDP629-20, IBIDP630-20, IBIDP632-20, IBIDP633-20, IBIDP641-20, IBIDP645-20, IBIDP682-20, IBIDP690-20, IBIDP710-20, IBIDP845-20	BOLD:AEC7677	MZ196916, MZ196669, MZ196789, MZ196718, MZ196618, MZ196809, MZ196523, MZ196875, MZ196752, MZ196604, MZ196732, MZ196719, MZ196750, MZ196647, MZ196572, MZ196698, MZ196531, MZ196562, MZ196790
Limoniidae (Limnophilinae)	*Dicranophragmanemorale* (Meigen, 1818) *	INV06199, INV06201	IBIDP256-19, IBIDP257-19	BOLD:AEC9486	MZ196708, MZ196735
Limoniidae (Limnophilinae)	*Hexatomanigra* Latreille, 1809 *	INV06356, INV06477, INV06478, INV06479, INV09307	IBIDP279-19, IBIDP285-19, IBIDP286-19, IBIDP287-19, IBIDP750-20	BOLD:AEC8914	MZ196712, MZ196815, MZ196814, MZ196563, MZ196799
Limoniidae (Limnophilinae)	*Hexatomaobscura* (Meigen, 1818) *	INV08133	IBIDP429-19	BOLD:AEC6596	MZ196917
Limoniidae (Limnophilinae)	*Paradelphomyiasenilis* (Haliday, 1833) "	INV08136	IBIDP432-19	BOLD:ADZ6740	MZ196564
Limoniidae (Limnophilinae)	*Euphylidoreaaperta* (Verrall, 1887) *	INV08529	IBIDP621-20	BOLD:AEE5475	MZ196717
Limoniidae (Limnophilinae)	*Phylidoreaferruginea* (Meigen, 1818)	INV06899, INV08137, INV08651, INV08998, INV09310, INV10294	IBIDP318-19, IBIDP433-19, IBIDP643-20, IBIDP707-20, IBIDP751-20, IBIDP819-20	BOLD:ABW4832	MZ196891, MZ196720, MZ196541, MZ196856, MZ196713, MZ196805
Limoniidae (Limnophilinae)	*Pseudolimnophilaebullata* Starý, 1982 *	INV07686, INV08122	IBIDP340-19, IBIDP418-19	BOLD:ABW9444	MZ196734, MZ196583
Limoniidae (Limnophilinae)	*Pseudolimnophilalucorum* (Meigen, 1818) "	INV07149, INV07150, INV07162, INV07309, INV09780, INV10281	IBIDP324-19, IBIDP325-19, IBIDP328-19, IBIDP333-19, IBIDP777-20, IBIDP816-20	BOLD:ABA4227	MZ196655, MZ196721, MZ196876, MZ196559, MZ196635, MZ196580
Limoniidae (Limoniinae)	*Achyrolimoniadecemmaculata* (Loew, 1873) "	INV08126	IBIDP422-19	BOLD:ABU8822	MZ196691
Limoniidae (Limoniinae)	*Dicranomyiaaffinis* (Staeger, 1840) "	INV08387, INV08388, INV08389, INV08390	IBIDP366-19, IBIDP367-19, IBIDP368-19, IBIDP369-19	BOLD:AEC8483	MZ196877, MZ196751, MZ196808, MZ196561
Limoniidae (Limoniinae)	*Dicranomyiachorea* (Meigen, 1818)	INV08131	IBIDP427-19	BOLD:ABU9992	MZ196706
Limoniidae (Limoniinae)	*Dicranomyiadidyma* (Meigen, 1804) "	INV04553, INV04580, INV04591, INV04593, INV04661, INV06485, INV08015, INV08392, INV09304	IBIDP191-19, IBIDP192-19, IBIDP194-19, IBIDP196-19, IBIDP215-19, IBIDP288-19, IBIDP416-19, IBIDP473-19, IBIDP748-20	BOLD:ADX2241	MZ196627, MZ196883, MZ196661, MZ196551, MZ196683, MZ196608, MZ196748, MZ196783, MZ196745
Limoniidae (Limoniinae)	*Dicranomyiaeulaliae* Geiger & Starý, 1994 *	INV10336	IBIDP839-20	BOLD:AEE5290	MZ196702
Limoniidae (Limoniinae)	*Dicranomyiahamata* Becker, 1908 *	INV04550, INV04592, INV08135	IBIDP190-19, IBIDP195-19, IBIDP431-19	BOLD:ADW6546	MZ196736, MZ196576, MZ196652
Limoniidae (Limoniinae)	*Dicranomyialongicollis* (Macquart, 1846) *	INV08149, INV08391	IBIDP445-19, IBIDP370-19	BOLD:AEC9918	MZ196909, MZ196631
Limoniidae (Limoniinae)	*Dicranomyiamaderensis* (Wollaston, 1858) *	INV08359	IBIDP454-19	BOLD:AED0520	MZ196841
Limoniidae (Limoniinae)	*Dicranomyiamodesta* (Meigen, 1818)	INV07031, INV08130, INV08555, INV08582, INV09011, INV09013	IBIDP322-19, IBIDP426-19, IBIDP631-20, IBIDP635-20, IBIDP708-20, IBIDP709-20	BOLD:AAI1352	MZ196599, MZ196769, MZ196664, MZ196558, MZ196621, MZ196714
Limoniidae (Limoniinae)	*Dicranomyianovemmaculata* (Strobl, 1906) *	INV08124	IBIDP420-19	BOLD:AEC9490	MZ196578
Limoniidae (Limoniinae)	*Dicranomyiapatricia* Starý, 1982 *	INV08129	IBIDP425-19	BOLD:AEC7553	MZ196826
Limoniidae (Limoniinae)	*Dicranomyiapauli* Geiger, 1983 *	INV06870, INV06967, INV07596, INV07772, INV07809, INV09763, INV09764, INV09858, INV10264	IBIDP408-19, IBIDP320-19, IBIDP337-19, IBIDP348-19, IBIDP414-19, IBIDP775-20, IBIDP776-20, IBIDP782-20, IBIDP809-20	BOLD:AEC9051	MZ196577, MZ196625, MZ196741, MZ196629, MZ196739, MZ196630, MZ196553, MZ196662, MZ196864
Limoniidae (Limoniinae)	*Dicranomyiasericata* (Meigen, 1830) *	INV08001, INV08140	IBIDP350-19, IBIDP436-19	BOLD:ACR3244	MZ196537, MZ196913
Limoniidae (Limoniinae)	*Dicranomyiavicina* (Macquart, 1838) *	INV08360	IBIDP455-19	BOLD:AEC9533	MZ196758
Limoniidae (Limoniinae)	*Dicranoptychafuscescens* (Schummel, 1829) "	INV06696, INV06698, INV08007, INV08152, INV08500, INV08505, INV08606, INV08858, INV08917, INV08923, INV08927, INV08929, INV08933, INV08940, INV08944, INV08948, INV08951	IBIDP309-19, IBIDP311-19, IBIDP355-19, IBIDP448-19, IBIDP614-20, IBIDP617-20, IBIDP640-20, IBIDP694-20, IBIDP697-20, IBIDP698-20, IBIDP699-20, IBIDP700-20, IBIDP701-20, IBIDP702-20, IBIDP703-20, IBIDP704-20, IBIDP706-20	BOLD:ADZ3003	MZ196545, MZ196836, MZ196673, MZ196549, MZ196824, MZ196690, MZ196692, MZ196663, MZ196526, MZ196803, MZ196865, MZ196737, MZ196733, MZ196862, MZ196660, MZ196651, MZ196674
Limoniidae (Limoniinae)	*Geranomyiabivittata* Becker, 1908 *	INV08362	IBIDP457-19	BOLD:AEC9740	MZ196781
Limoniidae (Limoniinae)	*Geranomyiaunicolor* Haliday, 1833 *	INV07561, INV07806	IBIDP336-19, IBIDP347-19	BOLD:AED0251	MZ196884, MZ196762
Limoniidae (Limoniinae)	*Heliuscalviensis* Edwards, 1928 *	INV08123, INV08581	IBIDP419-19, IBIDP634-20	BOLD:AEC6944	MZ196624, MZ196568
Limoniidae (Limoniinae)	*Heliushispanicus* Lackschewitz, 1928 *	INV08141, INV08687, INV08808, INV08809	IBIDP437-19, IBIDP648-20, IBIDP849-20, IBIDP850-20	BOLD:AEC9798	MZ196677, MZ196592, MZ196772, MZ196586
Limoniidae (Limoniinae)	*Limoniahercegovinae* (Strobl, 1898) *	INV06357, INV06369, INV08132, INV09306, INV09721, INV09826, INV10280	IBIDP280-19, IBIDP282-19, IBIDP428-19, IBIDP749-20, IBIDP770-20, IBIDP779-20, IBIDP815-20	BOLD:AEC9714	MZ196911, MZ196845, MZ196903, MZ196726, MZ196589, MZ196609, MZ196623
Limoniidae (Limoniinae)	*Limoniamaculipennis* (Meigen & Wiedemann, 1818) *	INV06022, INV06181, INV06287	IBIDP235-19, IBIDP251-19, IBIDP276-19	BOLD:ADX7619	MZ196823, MZ196860, MZ196835
Limoniidae (Limoniinae)	*Limonianubeculosa* Meigen, 1804	INV07774, INV07775, INV07776, INV08134, INV08430, INV08446, INV08488, INV08498, INV08503, INV08506, INV08534, INV08662, INV08698, INV08699, INV08705, INV08706, INV08707, INV08708, INV08770, INV08772, INV09038, INV09270, INV10295, INV10340	IBIDP341-19, IBIDP342-19, IBIDP343-19, IBIDP430-19, IBIDP592-20, IBIDP599-20, IBIDP611-20, IBIDP613-20, IBIDP615-20, IBIDP618-20, IBIDP623-20, IBIDP646-20, IBIDP651-20, IBIDP652-20, IBIDP655-20, IBIDP656-20, IBIDP657-20, IBIDP658-20, IBIDP680-20, IBIDP681-20, IBIDP718-20, IBIDP737-20, IBIDP820-20, IBIDP841-20	BOLD:AAG8508	MZ196646, MZ196573, MZ196728, MZ196777, MZ196831, MZ196682, MZ196671, MZ196716, MZ196672, MZ196528, MZ196894, MZ196633, MZ196641, MZ196616, MZ196648, MZ196700, MZ196869, MZ196696, MZ196797, MZ196880, MZ196878, MZ196544, MZ196581, MZ196870
Limoniidae (Limoniinae)	*Limoniaphragmitidis* (Schrank, 1781)	INV08441, INV08442, INV08452, INV08487, INV08738	IBIDP595-20, IBIDP596-20, IBIDP600-20, IBIDP610-20, IBIDP665-20	BOLD:ABV3744	MZ196782, MZ196850, MZ196775, MZ196556, MZ196761
Limoniidae (Limoniinae)	*Neolimoniadumetorum* (Meigen, 1804) "	INV08127, INV08472, INV08483, INV08485, INV08509, INV08547, INV08663, INV08697, INV08701, INV09018, INV09034, INV10221	IBIDP423-19, IBIDP605-20, IBIDP608-20, IBIDP609-20, IBIDP620-20, IBIDP627-20, IBIDP647-20, IBIDP650-20, IBIDP654-20, IBIDP711-20, IBIDP716-20, IBIDP792-20	BOLD:ABV5347	MZ196882, MZ196518, MZ196730, MZ196802, MZ196594, MZ196597, MZ196753, MZ196926, MZ196890, MZ196659, MZ196707, MZ196560
Pediciidae (Pediciinae)	*Pediciaocculta* (Meigen, 1830)	INV08003, INV08013	IBIDP590-20, IBIDP591-20	BOLD:AEE4508	MZ196888, MZ196725
Pediciidae (Pediciinae)	*Tricyphonaimmaculata* (Meigen, 1804)	INV08154,INV09303	IBIDP478-19, IBIDP747-20	BOLD:ADZ1801	MZ196759, MZ196524
Tipulidae (Ctenophorinae)	*Ctenophoraornata* Meigen & Wiedemann, 1818 *	INV05306, INV05449	IBIDP219-19, IBIDP222-19	BOLD:ADX2561	MZ196521, MZ196570
Tipulidae (Dolichopezinae)	*Dolichopezaalbipes* (Strom, 1768) "	INV00683	IBIDP587-20	BOLD:ACB7905	MZ196554
Tipulidae (Tipulinae)	*Nephrotomaappendiculatapertenua* Oosterbroek, 1978 "	INV04605, INV04614, INV06007, INV06172, INV06173, INV06177, INV06178, INV06192, INV06193, INV06275, INV06282, INV09272	IBIDP199-19, IBIDP208-19, IBIDP225-19, IBIDP246-19, IBIDP247-19, IBIDP248-19, IBIDP249-19, IBIDP254-19, IBIDP255-19, IBIDP267-19, IBIDP273-19, IBIDP738-20	BOLD:ADX1669	MZ196729, MZ196638, MZ196565, MZ196598, MZ196600, MZ196819, MZ196773, MZ196653, MZ196527, MZ196724, MZ196861, MZ196855
Tipulidae (Tipulinae)	*Nephrotomaflavipalpis* (Meigen, 1830) "	INV04862, INV05975, INV08700	IBIDP217-19, IBIDP392-19, IBIDP653-20	BOLD:ACZ3074	MZ196770, MZ196606, MZ196571
Tipulidae (Tipulinae)	*Nephrotomaguestfalica* (Westhoff, 1879) *	INV07242, INV08005, INV08713	IBIDP332-19, IBIDP353-19, IBIDP660-20	BOLD:ADS9890	MZ196857, MZ196821, MZ196895
Tipulidae (Tipulinae)	*Nephrotomaluteata* (Meigen, 1818) *	INV04296, INV04861, INV06546, INV09313, INV09314	IBIDP186-19, IBIDP216-19, IBIDP301-19, IBIDP752-20, IBIDP753-20	BOLD:ADW1410	MZ196522, MZ196574, MZ196536, MZ196649, MZ196800
Tipulidae (Tipulinae)	*Nephrotomasubmaculosa* Edwards, 1928 *	INV04526, INV04608, INV06006, INV06008, INV06009, INV06035, INV06171, INV06182, INV06272, INV08002, INV08379, INV08380, INV08480, INV08495, INV08504	IBIDP187-19, IBIDP202-19, IBIDP224-19, IBIDP226-19, IBIDP227-19, IBIDP243-19, IBIDP245-19, IBIDP252-19, IBIDP264-19, IBIDP351-19, IBIDP363-19, IBIDP364-19, IBIDP607-20, IBIDP612-20, IBIDP616-20	BOLD:AEC9536	MZ196620, MZ196693, MZ196686, MZ196615, MZ196829, MZ196763, MZ196812, MZ196786, MZ196642, MZ196844, MZ196754, MZ196617, MZ196710, MZ196899, MZ196887
Tipulidae (Tipulinae)	*Nephrotomasubmaculosa* Edwards, 1928 *	INV06021, INV06025, INV06170, INV06276	IBIDP234-19, IBIDP238-19, IBIDP244-19, IBIDP268-19	BOLD:ABW3498	MZ196552, MZ196667, MZ196816, MZ196588
Tipulidae (Tipulinae)	*Nephrotomasullingtonensis* Edwards, 1938 *	INV06270, INV06489, INV06497, INV09279, INV09295	IBIDP262-19, IBIDP290-19, IBIDP292-19, IBIDP742-20, IBIDP851-20	BOLD:ADX5268	MZ196668, MZ196595, MZ196534, MZ196839, MZ196704
Tipulidae (Tipulinae)	*Nephrotomasuturaliswulpiana* (Bergroth, 1888) *	INV07541	IBIDP335-19	BOLD:ABZ0908	MZ196922
Tipulidae (Tipulinae)	*Tipulacava* Riedel, 1913 *	INV06028, INV06030, INV09319, INV09320, INV09324	IBIDP239-19, IBIDP241-19, IBIDP757-20, IBIDP758-20, IBIDP762-20	BOLD:ADX7095	MZ196722, MZ196928, MZ196853, MZ196866, MZ196792
Tipulidae (Tipulinae)	*Tipulaconfusa* van der Wulp, 1883 "	INV09075, INV09076, INV10230, INV10245, INV10274, INV10333, INV10337, INV10343, INV10344, INV10349	IBIDP735-20, IBIDP736-20, IBIDP798-20, IBIDP803-20, IBIDP812-20, IBIDP838-20, IBIDP840-20, IBIDP843-20, IBIDP844-20, IBIDP846-20	BOLD:ABV4653	MZ196579, MZ196746, MZ196634, MZ196542, MZ196727, MZ196590, MZ196794, MZ196912, MZ196603, MZ196885
Tipulidae (Tipulinae)	*Tipulafabiola* Mannheims, 1968 *	INV08004, INV08764	IBIDP352-19, IBIDP678-20	BOLD:AEC9463	MZ196873, MZ196723
Tipulidae (Tipulinae)	*Tipulahelvola* Loew, 1873 *	INV08443	IBIDP597-20	BOLD:AAK1647	MZ196636
Tipulidae (Tipulinae)	*Tipulahispanolivida* Mannheims, 1968 *	INV05330	IBIDP220-19	BOLD:ADW8816	MZ196896
Tipulidae (Tipulinae)	*Tipulaiberica* Mannheims, 1963 *	INV04609, INV06278, INV06358	IBIDP203-19, IBIDP270-19, IBIDP281-19	BOLD:ADX7515	MZ196795, MZ196675, MZ196854
Tipulidae (Tipulinae)	*Tipulaintermedia* Eiroa, 1990 *	INV09057, INV10229, INV10254, INV10255, INV10259, INV10270, INV10287, INV10297, INV10309, INV10313	IBIDP732-20, IBIDP797-20, IBIDP805-20, IBIDP806-20, IBIDP808-20, IBIDP811-20, IBIDP818-20, IBIDP822-20, IBIDP829-20, IBIDP832-20	BOLD:ABZ5659	MZ196889, MZ196614, MZ196756, MZ196830, MZ196676, MZ196665, MZ196851, MZ196743, MZ196811, MZ196731
Tipulidae (Tipulinae)	*Tipulalateralis* Meigen, 1804 "	INV04630	IBIDP212-19	BOLD:ADK4356	MZ196575
Tipulidae (Tipulinae)	*Tipulalateralis* Meigen, 1804 "	INV05431, INV09056, INV10278, INV10286, INV10296, INV10300, INV10303, INV10316	IBIDP221-19, IBIDP731-20, IBIDP813-20, IBIDP817-20, IBIDP821-20, IBIDP824-20, IBIDP826-20, IBIDP834-20	BOLD:ABZ5659	MZ196678, MZ196897, MZ196904, MZ196801, MZ196550, MZ196847, MZ196567, MZ196879
Tipulidae (Tipulinae)	*Tipulamaxima* Pode, 1761 "	INV05298, INV06522, INV06532	IBIDP218-19, IBIDP299-19, IBIDP300-19	BOLD:AAD6106	MZ196923, MZ196622, MZ196687
Tipulidae (Tipulinae)	*Tipulamediterranea* Lackschewitz, 1830 *	INV04294, INV04613, INV04637, INV04660, INV06879, INV10187, INV10321	IBIDP185-19, IBIDP207-19, IBIDP213-19, IBIDP214-19, IBIDP314-19, IBIDP784-20, IBIDP835-20	BOLD:ADX2493	MZ196832, MZ196548, MZ196628, MZ196612, MZ196842, MZ196779, MZ196738
Tipulidae (Tipulinae)	*Tipulamorenae* Strob, 1900 *	INV06024, INV08010, INV08381	IBIDP237-19, IBIDP358-19, IBIDP365-19	BOLD:ADW6590	MZ196596, MZ196768, MZ196774
Tipulidae (Tipulinae)	*Tipulamorenae* Strob, 1900 *	INV06504, INV08014	IBIDP296-19, IBIDP361-19	BOLD:AEC7892	MZ196626, MZ196680
Tipulidae (Tipulinae)	*Tipulaoleracea* Linnaeus, 1758	INV06514, INV06929, INV07536, INV08461, INV08720, INV08727, INV08728, INV08742, INV08743, INV08744, INV08745, INV08749, INV08787, INV08788, INV08791, INV08792, INV08795, INV08796, INV09032, INV09039, INV09043, INV09050, INV09051, INV09052, INV09053, INV09055, INV10209	IBIDP298-19, IBIDP319-19, IBIDP334-19, IBIDP602-20, IBIDP661-20, IBIDP663-20, IBIDP664-20, IBIDP666-20, IBIDP667-20, IBIDP668-20, IBIDP669-20, IBIDP670-20, IBIDP684-20, IBIDP685-20, IBIDP686-20, IBIDP687-20, IBIDP688-20, IBIDP689-20, IBIDP715-20, IBIDP719-20, IBIDP722-20, IBIDP726-20, IBIDP727-20, IBIDP728-20, IBIDP729-20, IBIDP730-20, IBIDP786-20	BOLD:AAF9041	MZ196810, MZ196915, MZ196644, MZ196658, MZ196688, MZ196771, MZ196793, MZ196780, MZ196834, MZ196611, MZ196532, MZ196705, MZ196924, MZ196868, MZ196817, MZ196893, MZ196907, MZ196852, MZ196766, MZ196519, MZ196867, MZ196703, MZ196740, MZ196886, MZ196767, MZ196798, MZ196921
Tipulidae (Tipulinae)	*Tipulapaludosa* (Meigen, 1830) "	INV06872, INV06885, INV06889, INV09761, INV10219, INV10223, INV10332, INV10341	IBIDP313-19, IBIDP316-19, IBIDP317-19, IBIDP774-20, IBIDP790-20, IBIDP794-20, IBIDP837-20, IBIDP842-20	BOLD:ADZ7173	MZ196701, MZ196828, MZ196547, MZ196697, MZ196910, MZ196755, MZ196619, MZ196530
Tipulidae (Tipulinae)	*Tipulaparallela* Theischinger, 1977 *	INV04544, INV04615, INV06011, INV06029, INV06033, INV06273, INV06274, INV06277, INV09019, INV09273, INV09282, INV09293	IBIDP189-19, IBIDP209-19, IBIDP229-19, IBIDP240-19, IBIDP242-19, IBIDP265-19, IBIDP266-19, IBIDP269-19, IBIDP712-20, IBIDP739-20, IBIDP740-20, IBIDP741-20	BOLD:ADV9772	MZ196929, MZ196699, MZ196925, MZ196584, MZ196914, MZ196645, MZ196920, MZ196749, MZ196694, MZ196898, MZ196905, MZ196569
Tipulidae (Tipulinae)	*Tipulapilicauda* Pierre, 1922 *	INV06010, INV08012, INV08763	IBIDP228-19, IBIDP360-19, IBIDP677-20	BOLD:ADW9682	MZ196918, MZ196757, MZ196760
Tipulidae (Tipulinae)	*Tipulapseudocinerascens* Strobl, 1906 *	INV04527, INV04600, INV04606, INV04607, INV04610, INV04611, INV04616	IBIDP188-19, IBIDP197-19, IBIDP200-19, IBIDP201-19, IBIDP204-19, IBIDP205-19, IBIDP210-19	BOLD:ADX3916	MZ196871, MZ196742, MZ196863, MZ196881, MZ196849, MZ196813, MZ196695
Tipulidae (Tipulinae)	*Tipulapustulata* Pierre, 1920 *	INV08008	IBIDP356-19	BOLD:AEC8062	MZ196900
Tipulidae (Tipulinae)	*Tipularepanda* Loew, 1864 *	INV06880, INV07163, INV07164, INV10210, INV10217, INV10218, INV10220, INV10224, INV10235, INV10251, INV10298	IBIDP315-19, IBIDP329-19, IBIDP330-19, IBIDP787-20, IBIDP788-20, IBIDP789-20, IBIDP791-20, IBIDP795-20, IBIDP801-20, IBIDP804-20, IBIDP823-20	BOLD:AEC7761	MZ196591, MZ196892, MZ196689, MZ196709, MZ196822, MZ196657, MZ196874, MZ196543, MZ196838, MZ196901, MZ196827
Tipulidae (Tipulinae)	*Tipularufina* Meigen, 1818 "	INV10228	IBIDP796-20	BOLD:ACR4602	MZ196796
Tipulidae (Tipulinae)	*Tipulaintermixta* Riedel, 1913 *	INV06863	IBIDP312-19	BOLD:AEC7837	MZ196825
Tipulidae (Tipulinae)	*Tipulaserrulifera* Alexander, 1962 *	INV09722	IBIDP771-20	BOLD:AEE5789	MZ196765
Tipulidae (Tipulinae)	*Tipulatrifasciculata* Strobl, 1900 *	INV06279, INV08000, INV08378	IBIDP271-19, IBIDP349-19, IBIDP362-19	BOLD:ADX0913	MZ196557, MZ196906, MZ196670
Tipulidae (Tipulinae)	*Tipulatrigona* Mannheims, 1966 *	INV08006, INV08009, INV08011, INV08438, INV08445, INV08460, INV08462, INV08463, INV08726, INV08755, INV08757, INV08758, INV08760, INV08761, INV09027, INV09047	IBIDP354-19, IBIDP357-19, IBIDP359-19, IBIDP593-20, IBIDP598-20, IBIDP601-20, IBIDP603-20, IBIDP604-20, IBIDP662-20, IBIDP672-20, IBIDP673-20, IBIDP674-20, IBIDP675-20, IBIDP676-20, IBIDP714-20, IBIDP723-20	BOLD:AEC7552	MZ196610, MZ196538, MZ196806, MZ196927, MZ196529, MZ196566, MZ196684, MZ196764, MZ196656, MZ196643, MZ196818, MZ196533, MZ196791, MZ196546, MZ196582, MZ196820
Tipulidae (Tipulinae)	*Tipulavernalis* Meigen, 1804 "	INV06345, INV06347	IBIDP277-19, IBIDP278-19	BOLD:AAD2491	MZ196540, MZ196666
Tipulidae (Tipulinae)	*Tipulavittata* Meigen, 1804 *	INV09301	IBIDP745-20	BOLD:AEE0656	MZ196679
Tipulidae (Tipulinae)	*Tipulayerburyi* Edwards, 1924 *	INV09325	IBIDP763-20	BOLD:AEE8951	MZ196848
Tipulidae (Tipulinae)	*Tipulazarcoi* Mannheims, 1967 *	INV04603, INV06020, INV06023, INV06269, INV06271, INV06280, INV06283, INV06284, INV08477	IBIDP198-19, IBIDP233-19, IBIDP236-19, IBIDP261-19, IBIDP263-19, IBIDP272-19, IBIDP274-19, IBIDP275-19, IBIDP606-20	BOLD:ADX0912	MZ196784, MZ196654, MZ196846, MZ196711, MZ196637, MZ196785, MZ196602, MZ196535, MZ196650

**Table 2.** Number of specimens and species collected per Portuguese District or Autonomous Region and corresponding percentage.

**District or Region**	**Specimens (n)**	**Specimens (%)**	**Taxa (n)**	**Taxa (%)**
Bragança	88	21.4	35	42.2
Faro	41	10.0	29	34.9
Castelo Branco	38	9.2	19	22.9
Porto	35	8.5	15	18.1
Vila Real	33	8.0	14	16.9
Guarda	32	7.8	20	24.1
Setúbal	27	6.6	10	12.0
Santarém	23	5.6	11	13.3
Beja	20	4.9	9	10.8
Leiria	17	4.1	9	10.8
Coimbra	16	3.9	9	10.8
Lisboa	16	3.9	5	6.0
Viana do Castelo	12	2.9	7	8.4
Aveiro	7	1.7	4	4.8
Madeira	5	1.2	5	6.0
Azores	1	0.2	1	1.2
No data	1	0.2	1	1.2
**TOTAL**	**412**		**83**	
